# Cenobamate in refractory epilepsy: Overview of treatment options and practical considerations

**DOI:** 10.1002/epi4.12830

**Published:** 2023-10-03

**Authors:** Bettina Schmitz, Simona Lattanzi, Kristl Vonck, Reetta Kälviäinen, Lina Nashef, Elinor Ben‐Menachem

**Affiliations:** ^1^ Center for Epilepsy, Department for Neurology Vivantes Humboldt‐Klinikum Berlin Germany; ^2^ Neurological Clinic, Department of Experimental and Clinical Medicine Marche Polytechnic University Ancona Italy; ^3^ Department of Neurology, 4Brain Ghent University Hospital Gent Belgium; ^4^ Kuopio Epilepsy Center, Kuopio University Hospital, Member of ERN EpiCARE, and Institute of Clinical Medicine University of Eastern Finland Kuopio Finland; ^5^ Neurology Department King's College Hospital London UK; ^6^ Institution for Clinical Neuroscience, Sahlgrenska Academy University of Goteborg Goteborg Sweden

**Keywords:** anti‐seizure medications, cenobamate, drug resistant epilepsy, epilepsy polytherapy, epilepsy surgery, ketogenic diet, neurostimulation

## Abstract

Management of drug resistant epilepsy (DRE) represents a challenge to the treating clinician. This manuscript addresses DRE and provides an overview of treatment options, medical, surgical, and dietary. It addresses treatment strategies in polytherapy, then focuses on the role cenobamate (CNB) may play in reducing the burden of DRE while providing practical advice for its introduction. CNB is a recently approved, third generation, anti‐seizure medication (ASM), a tetrazole‐derived carbamate, thought to have a dual mechanism of action, through its effect on sodium channels as well as on GABA_A_ receptors at a non‐benzodiazepine site. CNB, having a long half‐life, is an effective add‐on ASM in refractory focal epilepsy with a higher response rate and a higher seizure‐freedom rate than is usually seen in regulatory clinical trials. Experience post‐licensing, though still limited, supports the findings of clinical trials and is encouraging. Its spectrum of action in relation to generalized epilepsies and seizures remains to be established, and there are no data on its efficacy in monotherapy. At the time of writing, CNB has been prescribed for some 50 000 individuals with DRE and focal epilepsy. A larger number is needed to fully establish its safety profile. It should at all times be introduced slowly to minimize the risk of serious allergic drug reactions. It has clinically meaningful interactions which must be anticipated and managed to maximize tolerability and likelihood of successful treatment. Despite the above, it may well prove to be of major benefit in the treatment of many patients with drug resistant epilepsy.

**Key Points**

- Cenobamate (CNB) is thought to have a dual mechanism of action acting on sodium channels and GABA_A_ receptors at a non‐benzodiazepine site.
- Adjunctive CNB was associated with higher rates of seizure response and seizure freedom than usually seen in regulatory trials.
- Post‐licensing experience, though still limited, supports the findings of clinical trials and is encouraging.
- The spectrum of action of CNB in generalized seizures and epilepsies remains to be established.
- Cenobamate may well prove to be of major benefit in the treatment of many patients with drug resistant epilepsy.

## 1 INTRODUCTION

Epilepsy represents one of the most common and serious neurological conditions, characterized by recurrent spontaneous seizures affecting about 70 million people worldwide.^1^ According to the International League against Epilepsy (ILAE) classification, seizure onset is classified as focal, generalized, and unknown and epilepsy as focal, generalized, or a combination of the two.^2^ Preventing seizures is the aim of every neurologist. Yet, anti‐seizure medications (ASMs) help achieve this in only up to two thirds of those affected with long‐lasting good seizure control.^3^, ^4^, ^5^, ^6^, ^7^, ^8^, ^9^, ^10^, ^11^, ^12^ Unfortunately, the remaining third or more still suffer from recurrent seizures despite ASMs, often prescribed in combination. Medical management of drug resistant epilepsy (DRE) represents a challenge to the treating clinician. This paper addresses DRE and provides an overview of treatment options. It then focuses on the role cenobamate (CNB) may play in reducing the burden of DRE, in addition to providing practical advice for its introduction.

## 2 DEFINITION OF DRUG RESISTANT EPILEPSY, RISK FACTORS AND PREDICTORS

Different criteria have been used to define DRE by both clinicians and researchers. The ILAE task force published a consensus definition of DRE, which can be regarded as a consensus opinion to be tested and refined as new evidence emerges. Its primary goal was to improve the care of patients and further clinical research. ILAE defined DRE as the failure of adequate trials of two tolerated and appropriately chosen and used ASM schedules (as monotherapy or in combination) to achieve sustained seizure freedom.^7^ Finnish national guidelines propose a wider definition of DRE identifying this as “Severe Epilepsy” and defining it as a state where the patient, despite adequate drug treatment, still experiences disabling symptoms interfering with daily life. These symptoms can be seizures but also adverse effects, cognitive problems or developmental delay in children.^8^

Achieving seizure freedom or a worthwhile reduction in seizure frequency for patients with DRE is often challenging. Some attain remission after several years and several drug trials. In a paper by Neligan et al., the authors identified patterns of remission and relapse in chronic DRE. Patients were classified into a number of pathways: “Pathway a”, which accounts for about 60%, includes patients achieving long‐term (probably permanent) remission within 5 years from diagnosis. “Pathway b”, comprising approximately 40% of the total population, includes those who still suffer from active epilepsy 5 years from diagnosis. Amongst these, some will experience subsequent long‐term remission (“Pathway c”). “Pathway d” includes those who continue to have epilepsy with no remission, while those with an intermittent pattern are included in “Pathway e”. The authors estimated that 10% of epilepsy patients overall follow an intermittent pattern and 20% are affected by a non‐remitting continuous epilepsy.^9^

Risk factors for developing DRE have been widely investigated and discussed. A major predictor of drug resistant epilepsy is the epilepsy syndrome.^10^, ^11^ A systematic review by Xue‐Ping Wang, published in 2019, sought to identify factors better able to predict drug resistance in patients with newly diagnosed epilepsy. The review identified abnormal electroencephalography (EEG) with both slow waves and epileptiform discharges, status epilepticus, focal onset seizures, symptomatic etiology, multiple seizure types, and febrile seizures as strong risk factors for developing DRE. The review did not confirm age of onset as a risk factor for DRE. Due to heterogeneity of study results, the authors could not draw firm conclusions for poor short‐term outcomes of therapy, neurodevelopment delay and high initial seizure frequency as predictive risk factors for DRE. This meta‐analysis also provided a quantitative estimate of the magnitude of the association between DRE and various risk factors.^12^

Several attempts have been made to improve prediction of DRE with a variety of both predictive models and biomarkers proposed. A recent review of potential clinical and biochemical markers for the prediction of drug resistant epilepsy rated the level of evidence for potential clinical predictors and biomarkers. The authors concluded that there is strong evidence that mixed seizure type, status epilepticus, poor response to the first ASM, neonatal seizures, abnormal findings on neuroimaging, and abnormal neurological examination are predictors of DRE. Of the potential biomarkers, they rated evidence as strong for raised HMGB1 (High mobility group box 1), a chromatin‐binding protein regulating gene transcription, and certain SCN1A gene polymorphisms.^13^

Looking at childhood onset epilepsy, an interesting prospective very long‐term population‐based study of 144 patients with epilepsy presenting in childhood before the age of 16 years followed up patients for an average of 37 (range 11–42) years. Overall, half the patients entered early or late remission without relapse and a further fifth entered remission after relapse. One‐third had poor long‐term outcome with persistent seizures after remission or without ever having entered remission.^14^

## 3 MANAGEMENT OF DRUG RESISTANT EPILEPSY

The goal of the treatment of epilepsy is to ensure the best possible quality of life by maximizing seizure control and minimizing side effects of epilepsy therapy. The real‐life everyday challenge is to identify the best treatment option for each patient.

### 3.1 Comparative effects of anti‐seizure medications

Evidence from head‐to‐head comparative studies of ASMs in DRE is very limited and comparisons are usually extrapolated from monotherapy studies, which are few and often designed to show non‐inferiority. Two pragmatic trials (SANAD and SANAD II) on the comparative effectiveness of ASMs in focal and in generalized epilepsies compared ASMs as first line agents and were not double‐blind.^15^, ^16^ There is only one, randomized, double‐blind, comparative study in drug resistant patients, a non‐inferiority trial to assess the efficacy and safety of levetiracetam, and pregabalin as adjunctive treatments in adult patients with focal seizures, inadequately controlled with at least two, and no more than five, prior ASMs. Pregabalin was noninferior to levetiracetam with respect to the primary endpoint of the proportion of patients with a ≥50% reduction in 28‐day seizure rate during the 12‐week maintenance phase as compared with baseline and had similar tolerability. There were no significant differences between pregabalin and levetiracetam for any of the secondary efficacy endpoints, including change in seizure frequency, proportion of secondarily generalized tonic–clonic seizure responders, and proportion of patients seizure free for the final 28 days of the study. In a post‐hoc analysis, the proportion of patients who were seizure free over the entire duration of the maintenance phase was lower with pregabalin (8.4%) than levetiracetam (16.2%) (*P* = 0.0155).^17^

### 3.2 From monotherapy to rational polytherapy, tolerability, and drug interactions

Kwan and Brodie^3^, ^4^ report that about 50% of epilepsy patients respond well to the initial monotherapy. Patients affected by DRE are usually treated with two or more different drugs. Rational polytherapy aims at both optimizing efficacy and minimizing side effects.^18^ Furthermore, the availability of third‐generation ASMs gave clinicians the options of trying different combinations of ASMs in non‐responders. Interactions between drugs is an important consideration when patients are on polytherapy, in relation to both anti‐seizure medications used in combination or non‐anti‐seizure medications, prescribed for comorbidities. When two drugs are given together, they can be synergistic (supra‐additive), additive, antagonistic (infra‐additive), or indifferent. When two drugs interact in an additive manner, their efficacy will be equal to the sum of their individual efficacy; when two drugs are given together and their combined efficacy is greater than the sum of their individual efficacy, the interaction is presumed to be supra‐additive. An infra‐additive effect can be seen when combined efficacy is reduced compared to the sum of the efficacy of each drug (Table 1).^19^

**TABLE 1:** Main pharmacodynamic interactions between antiseizure medications.^19^

Drug combination	Anti‐seizure activity	Neuro‐toxicity
VPA + ETX	Additivity	Infra‐additivity
CBZ + VPA	Additivity	Infra‐additivity
PHT + PB	Additivity	Infra‐additivity
PB + VPA	Additivity	Additivity
CBZ + PB	Additivity	Additivity
VPA + PHT	Supra‐additivity	Additivity
VPA + CLZ	Supra‐additivity	Supra‐additivity
ESM + CLZ	Supra‐additivity	Supra‐additivity
LTG + VPA	Supra‐additivity	Infra‐additivity
LTG + PB	Supra‐additivity	Supra‐additivity
LTG + CBZ	Infra‐additivity	Additivity
OXC + PHT	Infra‐additivity	Additivity
GBP + CBZ	Supra‐additivity	Additivity
GBP + VPA	Supra‐additivity	Additivity
GBP + PHT	Supra‐additivity	Additivity
TPM + LTG	Supra‐additivity	Infra‐additivity
TPM + OXC	Supra‐additivity	Additivity
TPM + GBP	Supra‐additivity	Additivity
TPM + FBM	Supra‐additivity	Infra‐additivity
GBP + OXC	Supra‐additivity	No toxicity
TGB + OXC	Additivity	Infra‐additivity
PGB + LTG	Additivity	Additivity
PGB + OXC	Additivity	Additivity
PGB + TPM	Additivity	Additivity
LCM + TPM	Supra‐additivity	No toxicity
LCM + LTG	Supra‐additivity	No toxicity
LEV + LCM	Supra‐additivity	No toxicity
LTG + OXC	Infra‐additivity	Supra‐additivity
OXC + FBM	Infra‐additivity	Additivity

There are no specific clinical guidelines regarding polytherapy, only largely theoretical recommendations often referred to as “rational polytherapy.”^20^ In general, the associations of ASMs characterized by different mechanisms of action (MoA) can have a favorable therapeutic index (supra‐additive for seizure protection and infra‐additive for neurotoxicity). Conversely, the combinations of ASMs with comparable MoA (eg, carbamazepine and lamotrigine, lamotrigine and oxcarbazepine, oxcarbazepine and phenytoin) are mainly characterized by infra‐additive efficacy and supra‐additive or additive neurotoxicity. The co‐administration of a single mechanism ASM and a second with multiple MoA may be advantageous.^19^ As confirmed in several clinical trials, combinations of drugs with sodium‐channel blocking effects led to a higher incidence of adverse events and withdrawal from the study than combinations of a sodium‐channel blocker and a drug with a different MoA.^21^, ^22^, ^23^

Beside efficacy, tolerability is another key aspect when combining multiple ASMs in patients affected by DRE. Tolerability has been demonstrated to be more related to drug load than the number of drugs taken. Drug load is defined as the ratio between the prescribed daily dose of a compound and the defined daily dose, the assumed average maintenance dose per day for a drug used for its main indication in adults.^24^, ^25^, ^26^ A higher drug load is associated with common central nervous system (CNS) adverse events, such as somnolence, dizziness, gait and balance disturbances, fatigue, blurred vision, and cognitive impairment. ASMs can also have rare, but serious idiosyncratic side effects such as Stevens‐Johnson syndrome/toxic epidermal necrolysis or drug reaction with eosinophilia and systemic symptoms (DRESS). Of note, some ASMs are associated with a higher risk of Stevens‐Johnson syndrome/toxic epidermal necrolysis, in particular the aromatic drugs carbamazepine, phenytoin, phenobarbital, and lamotrigine.^27^, ^28^ Other ASMs associated to a lesser extent with these idiosyncratic reactions include zonisamide, rufinamide, clorazepate, valproic acid, eslicarbazepine, oxcarbazepine, clonazepam, and levetiracetam.^28^

When combining therapy, and depending on the pre‐existing drug load, reducing the dose of the pre‐existing ASM can be a helpful strategy.^29^ Where side effects emerge, some authors suggest adjusting the dose of the existing drug rather than reducing the dose of the new ASM. In addition, both pharmacodynamic and pharmacokinetic characteristics of the drugs need to be considered. Some ASMs can induce or inhibit CYP enzymes, thus affecting the serum levels of concomitant drugs or their active metabolites.^30^, ^31^ Last but not least, an approach guided by the principle of “*start low and go slow*” is appropriate in many cases.^29^, ^30^

Fundamental to drug management in the treatment of epilepsy, especially with DRE patients, is to tailor treatment to patients' needs. The clinician may rely on guidelines while individualizing treatment on the basis of a multidimensional assessment of patients' individual social and clinical conditions, their priorities, characteristics of their epilepsy, drug pharmacokinetics, and pharmacodynamics. The objective should be finding the “drugs best fitting my patients”.^20^

In this regard, advances in the understanding of the mechanisms underlying epilepsies have enabled the identification of precision therapies that target specific etiologies. The main types of targeted treatments include: (1) substitutive therapies, currently used to treat epilepsies related to hereditary metabolic diseases (eg, ketogenic diet in epilepsy caused by GLUT1 deficiency syndrome); (2) therapies that modify cell‐signaling pathways, used to treat epilepsies related to the mTOR pathway and autoimmune epilepsy; (3) function‐based therapies that modify the function of ion channels, used to treat epilepsies caused by pathogenic variants resulting in gain or loss of function of these channels (eg, carbamazepine, oxcarbazepine, phenytoin, or lamotrigine in developmental and epileptic encephalopathies due to pathogenic gain‐of‐function variants in *SCN1A*).^32^

### 3.3 Non‐pharmacological treatment options

As soon as DRE or severe epilepsy is diagnosed, a referral to a specialized epilepsy center with dedicated diagnostic investigations such as optimum MRI and video‐EEG monitoring is mandatory for re‐evaluation of the diagnosis and optimal clinical management.^33^ Treatment modalities for patients with DRE include epilepsy surgery, neurostimulation, and dietary approaches. All options have specific advantages and drawbacks with regard to outcome and side effects and are typically used alongside ongoing and optimized ASM regimens. A recent meta‐analysis demonstrated that patients who underwent epilepsy surgery have a two‐fold lower risk of death and a three‐fold lower risk of SUDEP, but limited data are available for patients undergoing neuromodulation.^34^

#### 3.3.1 Epilepsy surgery

Different types of surgical procedures are currently available to control DRE including curative resective surgery (temporal lobectomy, selective amygdalohippocampectomy, extratemporal resection, hemispherectomy) and more palliative disconnective surgical procedures such as corpus callosotomy, multiple subpial transections (MST), and hemispherotomy. To establish suitability for epilepsy surgery and the most optimal surgical approach, patients with DRE are included in a presurgical evaluation protocol.^35^ The type of surgery that can or should be applied is highly patient and seizure type‐specific. In two randomized controlled trials (RCTs), superiority in efficacy and QOL for temporal lobe resections versus continued medical treatment was clearly demonstrated with seizure‐freedom rates of 58%–73%.^36^, ^37^ Variables associated with positive outcome after epilepsy surgery have been extensively investigated.^38^ The presence of a discrete resectable lesion and hippocampal sclerosis as etiology are associated with the highest seizure‐freedom rates. The investigation of the effectiveness and potential added value of less invasive epilepsy surgery approaches such as laser interstitial thermal therapy (LITT), thermocoagulation, and focused ultrasound is ongoing.^39^

#### 3.3.2 Neurostimulation

In many instances, patients are not suitable candidates for epilepsy surgery due to the absence of a unique and well‐defined epileptogenic zone or due to the location of the epileptic zone in functional brain tissue. For these patients, the available neurostimulation therapies or dietary treatments represent valuable add‐on options. *Vagus nerve stimulation*, approved since 1997, is the least invasive of the currently available neurostimulation therapies. The latest device incorporates a closed‐loop stimulation feature based on cardiac changes associated with seizure occurrence.^40^ After long‐term treatment, open studies report responder rates of >50% both in adults and children and both for focal and generalized seizures. Less than 10% of patients can be expected to become seizure free.^41^ VNS has been tried in refractory and super‐refractory status epilepticus with interruption of status reported in up to 74%, evidence from class IV studies and notification of a high risk of reporting bias.^42^ VNS is usually well tolerated with the most important side effects being stimulation‐related like hoarseness, coughing, and voice alternations. These are generally mild to moderate, can be reduced by adjusting the stimulation parameters and seldom necessitate the removal of the device.^43^ Moreover, positive effects on mood and quality of life have been reported in patients treated with VNS.^44^ *Deep brain stimulation* is a more recently developed neurostimulation treatment modality for selected patients with epilepsy delivering electrical current directly to the brain. Intracranial electrodes, targeting specific structures, and a pulse generator are implanted. DBS of the anterior nucleus of the thalamus, ANT‐DBS, is approved since 2010 and long‐term trials demonstrate favorable efficacy and safety profiles although intracranial hemorrhage was reported.^45^ Long‐term follow‐up studies of up to 10 years demonstrate seizure frequency percent reduction of up to 75% with no outcome differences related to prior vagus nerve stimulation or resective surgery. Responsive neurostimulation was approved in 2014 and has the unique feature of EEG recording capability, triggering intracranial stimulation in response to detected epileptiform EEG activity using implanted cortical strips and/or depth electrodes positioned at one or two seizure foci.^46^ Class 1 evidence from a randomized controlled trial and Class IV evidence from prospective open‐label follow‐up for a median of 8.97 years showed that responsive neurostimulation is acceptably safe, reduces seizure frequency, and improves quality of life.^47^ At 9 years in the open‐label study, the median percent reduction in seizures was 75% (responder rate 73%). Several less invasive neurostimulation and surgery approaches such as transcranial magnetic stimulation, transcranial direct current stimulation, and focused ultrasound are under investigation.^48^, ^49^, ^50^ With all approved neurostimulation therapies there are limitations on imaging, batteries will need replacing and lead failure may occur over time. The challenge for making these therapies more successful lies in the identification of responders to a specific type of neurostimulation, increasing knowledge on the mechanism of action, and development of personalized approaches to optimize stimulation parameters and clinical outcome. Analogous to the presurgical evaluation protocol for identification of the most suitable surgery approach for individual patients, efforts to develop a pre‐stimulation protocol are ongoing.^51^

#### 3.3.3 Dietary therapies

In 1921 Wilder proposed the ketogenic diet (KD) as a treatment for epilepsy. The aim of the dietary therapy is to mimic starvation, as studies in the past have shown anti‐seizure effects under such conditions.^52^ The diet encourages the intake of excessive amounts of fat and proposes daily meals containing 80% fat, 15% protein, and 5% carbohydrates. This is the so called 4:1 (fat: non‐fat) diet.^53^ In these circumstances, the body is forced to find an alternative energy source due to carbohydrate restriction. Stored body fat is transported to the liver where it is metabolized. Ketones are released into the circulation and used in the brain as an alternative energy source. The exact MoA of the KD in suppressing seizures is unknown. Ketogenic diet is a first line treatment for GLUT1 and pyruvate dehydrogenase deficiency. With regards to efficacy, a Cochrane review concluded an overall promising efficacy with high seizure‐freedom rates in very small children and seizure‐response rates of 75% but with less clear reliable effects in adults.^54^ Adverse events are mainly gastrointestinal (vomiting, diarrhea, and constipation). Hyperlipidemia, hypoglycemia, kidney stones, acute pancreatitis, cardiomyopathy, and death have been reported. Patients need to be closely monitored and long‐term follow‐up is desirable with intake of calcium, iron, and vitamin supplements. Successes have been reported in super‐refractory status epilepticus. Patients in intensive care units are often ketotic due to lack of nutrition in the initial stages making initiation of KD feasible in this setting.^55^ In recent years, the Modified Atkins' diet has been created as a more palatable and less restrictive dietary treatment for DRE.^56^ A recent prospective study investigated whether this modified Atkin's diet along with standard drug therapy was indeed more effective than standard therapy alone in adolescents and adults. At 6 months >50% seizure reduction was seen in 26.2% in the intervention group versus 2.5% in the control group (95% CI: 13.5–33.9; *P* < 0.001) with very few side effects.

### 3.4 Cenobamate and its pharmacology

CNB is a recently approved, third‐generation ASM, licensed for use as add‐on treatment for focal epilepsy in those aged 18 years or older. CNB is a novel tetrazole‐derived carbamate with a chiral center. While its MoA is not entirely understood, it is thought to have a dual MoA. It reduces neuronal excitability by promoting fast and slow inactivation of sodium channels and preferentially inhibiting the persistent component of the sodium channel current. In addition, it acts as a positive allosteric modulator of high affinity GABA_A_ receptors at a non‐benzodiazepine site.^57^, ^58^, ^59^ Based on data from rat hippocampal CA3 neurons it was also shown to have a higher affinity for inactivated compared to closed voltage‐gated sodium channels. Moreover, it was found that in rat hippocampal CA3 neurons, GABA currents were amplified by CNB in a concentration‐dependent manner.^60^ In preclinical studies, CNB has shown anti‐seizure activity in various rodent seizure models, namely electrical, chemical, and kindling models of both focal and generalized seizures, suggesting a broader spectrum of efficacy, possibly including generalized epileptic seizures.^61^ So far, only patients with focal seizures were included in regulatory trials. Reports of small series of patients with Lennox Gastaut and Dravet syndrome suggest efficacy beyond focal epilepsies.^61^, ^62^, ^63^ CNB suppressed intermittent photic stimulation induced photoparoxysmal response in patients with photosensitive epilepsy and was well tolerated in single doses up to 400 mg. The fact that most patients in this study also had a history of generalized epilepsy suggests that the photosensitivity model appears to screen for broad‐spectrum anti‐seizure activity.^64^

#### 3.4.1 Pharmacokinetics & drug–drug interactions

CNB pharmacokinetics are not linear in distribution or elimination. After multiple doses, the area under the concentration–time curve is proportional up to a dose of about 300 mg/day, but increases more than proportionally at higher doses. Its half‐life is about 55 h within the range of therapeutic doses (from 100 to 400 mg). Terminal half‐life, however, increases with increasing doses, from 30 h after a dose of 10 mg, up to 76 h after the highest tested dose of 750 mg. Accordingly, CNB clearance decreases at increasing doses.^65^ Protein binding is 60%. It undergoes complex hepatic metabolization with a final renal elimination.^66^ It has clinically relevant drug–drug interactions, and dose adjustments of other medication may be needed (Table 2).^67^ It can be an inducer or inhibitor of cytochrome enzymes. For example, CNB decreases the level of both carbamazepine and lamotrigine, while increasing the levels of phenytoin, clobazam (and its active metabolite), and phenobarbital. No clinically significant pharmacokinetic interactions have been reported when co‐administrated with valproate or lacosamide. There is no significant impact on plasma CNB with multiple‐dose exposure after repeated dosing of valproate, phenobarbital, and carbamazepine. Drug–drug interactions also occur with non‐ASMs (Table 3). For example, with oral contraceptives, the induction of CYP3A4 by CNB reduces estrogen and progestagen concentrations by more than 50%, resulting in possible failure of contraceptive therapy.^68^ Therefore, women of reproductive potential concomitantly using oral contraceptives should practice additional or alternative non‐hormonal measures of birth control. In a Phase I study no significant effect of CNB on warfarin concentrations was seen, indicating that CNB does not influence CYP 2C9, the primary pathway for warfarin metabolism. Porphyria was not addressed in interaction studies.^69^

**TABLE 2:** Pharmacology of Cenobamate: Mechanism of Action, Pharmacokinetics, Drug–Drug Interactions and Tolerability. Courtesy of Roberti et al. CNS Drugs 2021.^67^

	Effect on serum level	Antiseizure medication
CYP2C19‐Inhibition	Increase	Clobazam Phenytoin Phenobarbital Brivaracetam Cannabidiol Stiripentol
CYP3A4‐Induction	Decrease	Carbamazepine Clonazepam Perampanel Zonisamide Cannabidiol Stirpentol (Lacosamide)
Unspecific interactions/glucuronidation	Decrease	Lamotrigine (Levetiracetam)
No interactions	–	Gabapentin Pregabalin Oxcarbazepine Eslicarbazepine Rufinamide Topiramate Vigabatrin Valproate

**TABLE 3:** Drugs whose metabolism may be altered by cenobamate.

	Effect of the combination	Mechanism of interaction	Selection of drugs affected	Clinical comment
CYP3A4 substrates	↓ serum level	CYP3A4 induction	Itraconazole Methadone Nevirapine Sorafenib Tamoxifen	Risk for reduced efficacy, increase the dosage as needed
			Oral contraceptives	Additional or alternative types of birth control should be used
CYP2B6 substrates	↓ serum level	CYP2B6 induction	Bupropion Propofol Selegilin	Risk for reduced efficacy, increase the dosage as needed
CYP2C19 substrates	↑ serum level	CYP2C19 inhibition	Omeprazole Pantoprazole Sertraline	Risk for adverse events, reduce the dosage as needed
CNS depressants	Dose‐dependent adverse effects	Additive effect	Benzodiazepine (Diazepam) Barbiturates (Phenobarbital) Sedative hypnotics (Zolpidem)	These drugs should be prudently co‐administered
QT‐shortening drugs	Dose‐dependent QT‐shortening	Additive effect	β‐blockers (Propranolol) Rufinamide	These drugs should be prudently co‐administered

*Note*: Courtesy of Roberti et al.^67^ Pharmacology of Cenobamate: Mechanism of Action, Pharmacokinetics, Drug–Drug Interactions, and Tolerability. CNS Drugs 2021.

Abbreviations: CNS, central nervous system; CYP, cytochrome P 450.

### 3.5 Cenobamate: clinical trial data

Three clinical trials were undertaken as summarized (Table 4). The first two trials, C013 and C017,^70^, ^71^ were multicenter, randomized, double‐blind, placebo‐controlled clinical trials in uncontrolled focal epilepsy. The latter, C021^72^ was a large phase 3, multicenter, open‐label safety study. Trial C013 demonstrated that CNB, when titrated up to 200 mg, significantly improved seizure control by reducing seizure frequency in >50% of patients with a seizure‐freedom rate of 28.3% in the maintenance phase (6 weeks), while 8.8% of placebo‐treated patients were seizure free. In the completer population using an ITT approach that divides the number of seizure‐free completers by the intention‐to‐treat (ITT) population, 24.8% of patients (28 of 113) in the cenobamate group achieved seizure freedom during the maintenance phase compared with 8.3% (9 of 108) in the placebo group.

**TABLE 4:** Adverse event of adjunctive cenobamate versus placebo in randomized, controlled trials.^76^

Outcome	Number of events/participants (%)		Risk ratio (99% CI)	*p* value
	CNB	Placebo		
Any AE	340/442 (76.9)	115/217 (53.0)	1.14 (0.99–1.31)	0.021
Treatment‐related AE	293/442 (66.3)	96/217 (44.2)	1.46 (1.17–1.83)	<0.001
Any SAE	24/442 (5.4)	10/217 (4.6)	0.99 (0.36–2.75)	0.978
Somnolence	109/442 (24.7)	22/217 (10.1)	2.35 (1.31–4.24)	<0.001
Dizziness	103/442 (23.3)	33/217 (15.2)	1.53 (0.94–2.49)	0.026
Headache	49/442 (11.1)	20/217 (9.2)	1.27 (0.63–2.57)	0.374
Nausea	31/442 (7.0)	6/217 (2.8)	2.98 (0.92–9.61)	0.017
Fatigue	71/442 (16.1)	16/217 (7.4)	1.96 (0.97–3.95)	0.014
Nystagmus	25/442 (5.7)	1/217 (0.5)	7.83 (0.91–67.64)	0.014
Balance disorder	24/442 (5.4)	1/217 (0.5)	9.19 (1.04–80.96)	0.009
Upper respiratory infection	18/442 (4.1)	11/217 (5.1)	0.88 (0.34–2.29)	0.721
Constipation	21/442 (4.8)	1/217 (0.5)	6.71 (0.77–58.40)	0.024
Vomiting	17/442 (3.8)	2/117 (0.9)	3.64 (0.60–22.27)	0.066
Urinary tract infection	9/113 (8.0)	2/109 (1.8)	4.34 (0.60–31.56)	0.057
Tremor	7/113 (6.2)	3/109 (2.8)	2.25 (0.39–12.87)	0.231
Nasopharyngitis	7/113 (6.2)	1/109 (0.9)	6.75 (0.44–103.73)	0.072
Diarrhea	6/113 (5.3)	0/109 (0.0)	12.54 (0.29–541.21)	0.084
Anxiety	1/113 (0.9)	6/109 (5.5)	0.16 (0.01–2.54)	0.088
Ataxia	13/329 (4.0)	1/108 (0.9)	4.27 (0.30–60.87)	0.160
Dysarthria	12/329 (3.6)	0/108 (0.0)	8.26 (0.20–335.34)	0.142
Diplopia	36/329 (10.9)	2/108 (1.9)	5.91 (0.93–37.56)	0.013
Fall	10/329 (3.0)	6/108 (5.6)	0.55 (0.15–2.01)	0.232
Back pain	11/329 (3.3)	3/108 (2.8)	1.20 (0.23–6.29)	0.773
Vertigo	10/329 (3.0)	3/108 (2.8)	1.09 (0.21–5.82)	0.890
Decreased appetite	10/329 (3.0)	1/108 (0.9)	3.28 (0.22–48.19)	0.254
Gait disturbance	16/329 (4.9)	3/108 (2.8)	1.75 (0.36–8.63)	0.366

Abbreviations: AE, adverse event; CNB, cenobamate; CI, confidence interval; SAE, serious adverse event.

C017, which randomized patients to placebo, 100, 200, or 400 mg cenobamate, showed that with higher doses more patients discontinued their treatment because of an increased rate of treatment‐emergent adverse events (TEAE). The design of the trial was such that patients were not able to adjust the dosage of their pre‐existing ASM therapy when titrating to higher doses and this may have increased the incidence of TEAE. Of note, however, is that higher doses were associated with a higher response rate.

In the CO21 study patients received CNB as add‐on therapy at starting doses of 12.5 mg titrated over 10–12 weeks to a target of 200 mg with further allowed increases up to 400 mg. This study design allowed for reduction in concomitant ASMs, maintaining a steady state of plasma levels during CNB titration. The study showed a high retention rate in patients exposed to CNB for more than 6 months (82.9%). Adverse events were consistent with the two earlier studies (Table 5), but with the slower titration, no case of DRESS were observed. Whereas the target dose of this study was the same, the low initial dose and slower titration schedule may have influenced the findings. In general, the “start low and go slow” approach adopted in C021 appears to be the best for efficacy and tolerability of CNB. With a half‐life of 55 h (range 50–60 h at standard doses), it would take more than 10 days for CNB to reach a steady state and slow titration is essential to assess efficacy at a given dose, manage interactions, and minimize the risk of side effects.

**TABLE 5:** Cenobamate clinical trials.

	Randomized study C013^70^ NCT01397968	Randomized study C017^71^ NCT01866111	Open‐label safety study C021^72^ NCT02535091
Study design DB	12‐week, randomized, DB, PBO‐controlled study followed by possible OLE for participants who completed DB^a^ study periods	18‐week, randomized, DB, PBO‐controlled study followed by possible OLE for participants who completed DB study periods	―
Cenobamate target dose DB	200 mg/day	100, 200 or 400 mg per day	―
Titration schedule	Initial 50 mg/day up‐titrated by 50 mg/day every 2 weeks to 200 mg/day	Initial 50 mg/day up‐titrated by 50 mg/week to 200 mg, and then 100 mg/day weekly to 400 mg/day	―
Study design OLE/OL	Conversion to target OLE cenobamate dose followed by up to several years of OL treatment^b^	Conversion to target OLE cenobamate dose followed by 52 weeks of OL treatment and continuation for those who were benefiting^b^	12‐month OL with possible continuation for those who were benefiting
Cenobamate target dose OLE/OL	200 mg/day (maximum 400 mg/day)	300 mg/day (maximum 400 mg/day)	200 mg/day (maximum 400 mg/day)
Titration schedule OLE/OL	Initial 100 mg/day dose increased by 50 mg/week every 2 weeks to 200 mg/day; further increases up to 400 mg/day allowed	2‐week conversion to the target dose of 300 mg/day; further increases up to 400 mg/day, and decreases in the dose to 50 mg/day was allowed	Initial 12.5 mg/day up‐titrated every 2 weeks (25, 50, 100, 150, 200 mg/day) to 200 mg/day, then up‐titrated by 50 mg/day every 2 weeks, to maximum of 400 mg/day

Abbreviations: DB, double‐blind; OL, open‐label; OLE, open‐label extension; PBO, placebo.

^a^ With the exception of 43 participants participating at sites in India.

^b^ Or until development stopped by SK life science, Inc., or the product is approved for marketing, or anytime at the discretion of SK life science, Inc.

When given as adjunctive therapy, titrating up to 100, 200, or 400 mg per day, CNB demonstrated a significant decrease in baseline seizure frequency of 50% or higher compared to placebo. When titrated to a dosage of 200 and 400 mg, it lowered seizure frequency by 75%–90% or more, even reaching seizure freedom. Across the trials, treatment withdrawal was seen in 16.7% and 11.1% of participants receiving, respectively, CNB and placebo. Adverse events presented by patients undergoing therapy with CNB were CNS related including somnolence, dizziness, fatigue, balance disorders, and diplopia. They were reported to occur in 76.9% and in 66.8% of patients randomized, respectively, to add‐on CNB vs placebo. Four patients developed hypersensitivity, manifesting as skin reactions, and one developed DRESS (Table 6), including one fatality.^72^, ^73^, ^74^ These four participants started on CNB doses of 50 or 100 mg/day with weekly dose increases. A slow titration schedule with a lower initial dose and slow up‐titration to bi‐weekly lowered the risk of developing acute hypersensitivity reactions like DRESS (Table 5). A dose‐dependent, non‐clinically relevant shortening of QT interval (≤340 ms) was shown by CNB, the effect probably due to inhibition of cardiac sodium channels.^75^ Given this observation, CNB is contraindicated in patients with familial short QT syndrome, and caution is needed with other QT‐shortening drugs, as synergistic effects can exist. There was a trend towards hyperkalemia observed in 17% of treated patients compared to 7% on placebo, but no cardiac events related to this rise in blood level of potassium were recorded. These patients reported at least one value in the study over 5 mq/L but did not show consistently high potassium levels throughout the study.

**TABLE 6:** Treatment‐emergent adverse events reported during clinical trials with cenobamate.

	Randomized study C013^70^ NCT01397968		Randomized study C017^71^ NCT01866111				Open‐label safety study C021^72^ NCT02535091
	Cenobamate 200 mg/day	Placebo	Cenobamate 100 mg/day	Cenobamate 200 mg/day	Cenobamate 400 mg/day	Placebo	Cenobamate 200 mg/day
*N*	113	109	108	110	111	108	1339
Any TEAE, *n* (%)	86 (76.1)	69 (63.3)	70 (64.8)	84 (76.4)	100 (90.1)	76 (70.4)	1128 (84.2)
Treatment‐related TEAEs, *n* (%)	67 (59.3)	50 (45.9)	62 (57.4)	72 (65.5)	92 (82.9)	46 (42.6)	935 (69.8)
Serious TEAEs, *n* (%)	2 (1.8)	4 (3.7)	10 (9.3)	4 (3.6)	8 (7.2)	6 (5.6)	108 (8.1)
TEAEs ≥10%, *n* (%)							
Somnolence	25 (22.1)	13 (11.9)	20 (18.5)	23 (20.9)	41 (36.9)	9 (8.3)	376 (28.1)
Dizziness	25 (21.1)	18 (16.5)	19 (17.6)	22 (20.0)	37 (33.3)	15 (13.9)	316 (23.6)
Fatigue	12 (10.6)	7 (6.4)	13 (12.0)	19 (17.3)	27 (24.3)	9 (8.3)	222 (16.6)
Diplopia	–	–	8 (7.4)	11 (10.0)	17 (15.3)	2 (1.9)	78 (5.8)
Headache	14 (12.4)	14 (12.8)	11 (10.2)	12 (10.9)	12 (10.8)	6 (5.6)	152 (11.4)
Nausea	13 (11.5)	5 (4.6)	7 (6.5)	1 (0.9)	10 (9.0)	1 (0.9)	80 (6.0)

*Note*: In C013 and C017, TEAEs were reported during the double‐blind treatment period, and in C021, TEAEs were reported based on the data cut‐off of April 2018.

Abbreviations: PBO, placebo; TEAE, treatment‐emergent adverse event.

Thus, CNB efficacy in drug resistant focal epilepsy has been confirmed by two RCTs^70^, ^71^ as shown by the meta‐analysis published by Lattanzi.^76^ A network meta‐analysis by Lattanzi et al.,^77^ which included 16 RCTs, showed that CNB has statistically superior efficacy in terms of 50% responder rate over the comparators brivaracetam, lacosamide, perampanel, and eslicarbazepine acetate. Furthermore, even though no statistical significance was found, CNB was the ASM associated with the highest probability of seizure freedom compared to the comparators.

The seizure freedom rates for CNB and the comparators are shown in Table 7. The rates of seizure freedom were estimated for most trials by adopting the “pragmatic ITT” approach,^78^ whereby only patients that complete the study and are seizure free can be classed as seizure free in the numerator and the modified intention‐to‐treat (mITT) population (consisting of all randomized patients who received at least one dose of the study drug and had any seizure frequency data collected during the double‐blind phase) is used as denominator; in three studies (two for eslicarbazepine and one for perampanel), outcome up to treatment discontinuation was used to impute the seizure‐freedom status for the remainder of the trial.

**TABLE 7:** Seizure‐freedom rates with adjunctive third‐generation antiseizure medications in randomized, placebo‐controlled, trials.^77^

Antiseizure medication	Number of studies	Seizure‐freedom rate
Brivaracetam	3	3.9%
Cenobamate	1	11.8%
Eslicarbazepine acetate	4	4.1%
Lacosamide	4	3.1%
Perampanel	4	3.5%

*Note*: See text for details.

Seizure‐free rates were dose dependent in the CO17 trial^70^ with an increase in seizure‐free rates and efficacy with each increase in dose.^26^, ^76^ Seizure frequency reductions by 100% during the 12‐week maintenance phase were 1% for the placebo group, 4% for the CNB 100 mg group, 11% for the CNB 200 mg group, and 21% for the CNB 400 mg. Looking at these data, CNB seems to be the best option for efficacy, especially for patients with DRE. On the other hand, it may not be as tolerable at this dose since this paper reported a higher rate of TEAEs with CNB at 400 mg per day compared to CNB at 200 mg per day and compared to other compounds.

As it is licensed as add‐on therapy in focal epilepsy, patients being administered CNB will be already on other medications. Therefore, it is important to ensure patients are able to tolerate its introduction into a pre‐existing therapeutic regimen, with regular monitoring, slow titration, awareness of interactions, and adjustment/lowering of concomitant medication. Pharmacokinetic interactions usually arise earlier in the titration period mostly due to variations in cytochrome P450 isoenzymes. On the other hand, pharmacodynamic interactions, characterized by changes in pharmacological effects of a specific drug without variations in its plasma levels, are more likely to occur later in the titration period, when concomitant ASMs are at higher doses, and especially with combinations of drugs with the same MoA. These pharmacodynamic interactions are characterized by increased toxicity and infra‐additive efficacy.^19^ With proactive adjustments of the concomitant ASMs during titration, it is hoped that adverse events due to pharmacodynamic and pharmacokinetic interactions can be avoided.

To investigate this further, a post‐hoc analysis of the phase 3 open‐label study C021 was carried out by Rosenfeld et al. in 2021.^79^ The paper examined how dose adjustments of baseline concomitant ASMs, allowed in the C021 study, influenced tolerability, efficacy, and retention of CNB. The flexibility allowed in dose reduction in the C021 trial led to faster and earlier changes in doses of concomitant drugs. Phenytoin, phenobarbital, clobazam, valproate, and lacosamide were lowered earlier during the CNB up‐titration phase, while carbamazepine, oxcarbazepine, and eslicarbazepine were decreased later during the maintenance phase. Specific pharmacokinetic interactions leading to changes in drug exposure and tolerability may have underlined these differences. Phenobarbital and phenytoin are metabolized by the CYP2C19 isoenzyme which is inhibited by CNB. Their blood levels were found to be increased by a mean of 37% and 84% respectively when add‐on CNB was started. Phenobarbital, in this study, was proactively lowered during the CNB titration phase. Clinicians also need to be proactive about monitoring and reducing phenytoin, and this can be guided by the baseline blood level.^80^ TEAEs may also occur when patients taking clobazam are started on CNB, as CNB inhibits CYP2C19 enzyme. This enzyme metabolizes the active metabolite of clobazam (N‐desmethylclobazam), an increase in the blood level of which can lead to adverse events like somnolence. Thus, an early proactive reduction of clobazam was also recommended when starting CNB in this study.

Some recommendations that evolved from the above studies are that while high doses of lacosamide and CNB do not usually interact, at 400 mg per day of CNB, the inhibition of CYP2C19 can lead to a small increase in lacosamide levels. Thus, both pharmacokinetic and pharmacodynamic interactions can occur between these two medications.^79^ Other sodium channels blockers like carbamazepine, oxcarbazepine, eslicarbazepine, and lamotrigine are believed to not require dose reduction until patients are in CNB maintenance phase. They are metabolized by CYP3A4, which is induced by CNB, and the introduction of CNB can lower their blood levels. This effect can reduce the incidence of TEAEs while CNB is up‐titrated. Valproate (VPA) can be reduced in the late CNB titration phase, however, no drug–drug interactions have been described between VPA and CNB (package insert). There was no interaction between levetiracetam and CNB. This post‐hoc analysis^79^ therefore indicates that reducing concomitant ASMs dosage can result in a greater retention rate likely due to improved tolerability attributable to a reduced drug load. A recently published expert opinion consensus paper^80^ addressing the same topic provided recommendations for reducing the dose of concomitant ASMs during titration of CNB. The group distinguished between proactive and reactive dose adjustment, defining them as ones made prior to or following patient self‐reported adverse event, respectively. Proactive dose adjustment is reasonable where total drug load and predicted interactions with concomitant medications are likely to result in toxicity, that is when pharmacokinetic and/or pharmacodynamic interactions are expected to occur.

Since CNB has been tested only as add‐on therapy, pharmacodynamic interactions may have contributed to the reported neuropsychiatric TEAS. In the pivotal trials at baseline, 2.2% to 6.5% had memory impairment, 10.6% to 14.3% had depression, and 6.6%–11.1% had anxiety. During the trial the TEAS for cognitive side effects was less than 2% and for psychiatric side effects less than 3%.^77^

### 3.6 Berlin: 30 months experience with CNB

Published real‐0world experience with the use of cenobamate is limited but encouraging, with good response rates and reductions in concomitant medication.^81^, ^82^ In the epilepsy center at the Vivantes Humboldt Klinikum, Berlin, treatment with CNB was started in October 2020 in the context of a named patient programme. In May 2023 a cohort of 86 patients had a minimum follow‐up of 3 months. The median age of patients was 36 years, ranging from 19 until 83 years. The mean duration of epilepsy was 24 (4–61) years, and 40 patients were females. With the exception of four patients (one Lennox Gastaut syndrome, one Rett‐syndrome, and two Dravet syndrome), all patients suffered from focal onset seizures (38 with unknown, 44 with structural etiology). All patients were pharmaco‐resistant with a median number of 5 (range 2–15) previously tried ASMs. The median follow‐up was 19 (range 3–30) months. At the end of follow‐up, the majority of patients were on a daily dosage of 200 mg CNB (23%: 12.5–25 mg, 25%: 50–100 mg, 48%: 150–400 mg, 4%: 500 mg).

In 28 (33%) patients, CNB was withdrawn for the following reasons: side effects in 20 patients: exanthema without systemic symptoms (*n* = 3), seizure exacerbation (*n* = 3), depression (*n* = 3), gastrointestinal problems (*n* = 3), cognitive slowing (*n* = 2), unspecific complaints (*n* = 2), somnolence (*n* = 1), headache (*n* = 1), dizziness (*n* = 1), gait disturbance (*n* = 1). All side effects were reversible. One patient became pregnant (resulting in a healthy child). Lack of efficacy lead to withdrawal of CNB in seven cases. The responder rate was calculated based on the following definition: seizure reduction of at least 50% (compared to the year before CNB was started) or seizure freedom (defined as a seizure‐free period of at least three times the median seizure interval in the year before onset of CNB). Using this definition, the responder rate was 52%, including 36% of patients with a mean seizure reduction rate of ≥50% and 16% patients becoming seizure free (including one patient with Dravet syndrome). Another 10% of patients had their seizure frequency unchanged, 5% had an unclear seizure response, and withdrawal occurred in 33%.

These preliminary real‐world observations are in agreement with findings from regulatory trials and support a high efficacy in difficult to treat patients and a good tolerability of CNB.

## 4 CONCLUSION

CNB is an effective newly licensed add‐on ASM in refractory focal epilepsy. Its spectrum of action in relation to generalized epilepsies and seizures remains to be established, and there are no data on its efficacy in monotherapy. It has, at the time of writing, been prescribed for some −50 000 with DRE and focal epilepsy but a larger number is needed to fully establish its safety profile. It should at all times be introduced slowly to minimize the risk of serious drug allergies. Thus, it is not a medication for emergency use. It has clinically meaningful interactions which must be anticipated and managed to maximize tolerability and likelihood of successful treatment. It is currently only available in tablet formulation.

Despite the above, it has shown to be efficacious in DRE with a higher response rate and a higher seizure‐freedom rate than is usually seen in regulatory clinical trials. Experience post‐licensing, though still limited, supports the findings of clinical trials and is encouraging as shown by experience of one of the authors outlined above, with anecdotal positive experience also as a possible wide‐spectrum ASM for epileptic encephalopathies. It may well be a “game changer” in the treatment of many patients with drug resistant epilepsy.^83^ This paper summarizes available data on CNB in addition to providing practical advice for its use, where indicated, in DRE.

## AUTHOR CONTRIBUTIONS

Bettina Schmitz planned and designed the study, interpreted the data, drafted, and revised the manuscript. Simona Lattanzi planned and designed the study, interpreted the data, drafted, and revised the manuscript. Kristl Vonck planned and designed the study, interpreted the data, drafted, and revised the manuscript. Reetta Kälviäinen planned and designed the study, interpreted the data, drafted, and revised the manuscript. Lina Nashef planned and designed the study, interpreted the data, drafted, and revised the manuscript. Elinor Ben‐Menachem planned and designed the study, interpreted the data, drafted, and revised the manuscript. All authors approved the final submitted version.

## 5 CONFLICT OF INTEREST statement

Bettina Schmitz has received speaker's or consultancy fees from Angelini Pharma, Desitin, Eisai, Precisis, Sanofi, and UCB‐Pharma outside the submitted work. Simona Lattanzi has received speaker's or consultancy fees from Angelini Pharma, Eisai, GW Pharmaceuticals, and UCB Pharma and has served on advisory boards for Angelini Pharma, Arvelle Therapeutics, BIAL, Eisai, GW Pharmaceuticals, and Rapport Therapeutics outside the submitted work. Kristl Vonck reported receiving speaker/consulting fees from LivaNova Europe, Angelini Pharma, Pharvaris, Al Mann Foundation, Synergia Medical, and Precisis outside the submitted work. Reetta Kälviäinen has received speaker's or consultancy fees from Angelini Pharma, Eisai, Jazz Pharma, Orion, UCB Pharma, and Takeda outside the submitted work. Lina Nashef has received speaker's fees, advisory board fees and has been sponsored to attend medical meetings by industry including Angelini Pharma, GW Pharmaceuticals, Jazz pharmaceuticals, UCB Pharma, and LivaNova outside the submitted work. Elinor Ben‐Menachem received speaker's or consultancy fees from Angelini Pharma, Congiguard, Theracule, Xenon, and UCB Pharma outside the submitted work.

## ETHICS STATEMENT

We confirm that we have read the Journal's position on issues involved in ethical publication and affirm that this report is consistent with those guidelines.
